# Supplementary material for: Self-Medication and Safety Profile of Medicines Used among Pregnant Women in a Tertiary Teaching Hospital in Jimma, Ethiopia: A Cross-Sectional Study
Source: Int J Environ Res Public Health. 2020 Jun 4;17(11):3993. doi: 10.3390/ijerph17113993 (PMC7312933; doi:10.3390/ijerph17113993)
Supplement: Supplementary file 1 [file ijerph-17-03993-s001.pdf]

Table S1: Medicinal plants used along with self-medication among pregnant women prior to admission to JUMC, Ethiopia

**Type of Medicinal plants used (N=110)**	**Local name (English name) [a]**	**Number of users**	**Percent age [b]**
Linum usitatissimum L.	*Telba* (Flaxseed)	88	80.9
Zingiber officinale Roscoe.	*Zingibil* (Ginger)	15	13.6
Carica papaya L.	*Papaya* (Papaya)	14	12.7
Ocimum lamiifolium Hochst. ex Benth.	*Damakessie* (No English name) [c]	14	12.7
Allium sativum L.	*Nech shinkrut* (Garlic)	12	10.9
Trigonella foenum-graecum L.	*Abish* (Fenugreek)	7	6.4
Nigella sativa L	*Tikur Azmud* (Black seed)	7	6.4
Eucalyptus globulus Labill.	*Nech-bahir zaf* (''Eucalyptus'')	6	5.5
Ruta chalepensis L.	*Tenadam* (Fringed rue)	5	4.5
Cinnamomum verum J.Presl	*Qarafa* (Cinnamon)	3	2.7
Taverniera abyssinica A. Rich	*Dingetegna* (No English name)	2	1.8
Artemisia abyssinica Sch.Bip. ex A.Rich.	*Chikugn* (No English name)	1	0.9
Brassica nigra (L.) K.Koch	Senafitch (Black mustard)	1	0.9
Echinops kebericho Mesfin	*Kebericho* (No English name)	1	0.9
Hagenia abyssinica (Bruce ex Steud.) J.F.Gmel.	*Kosso* (African redwood)	1	0.9
Phoenix dactylifera L.	*Temir* (Dates)	1	0.9
Vernonia amygdalina Del.	*Grawa* (Bitter leaf)	1	0.9

[a]Local name in *Amharic* language; [b]Total percentage may exceed 100% due to multiple responses; [c]No English name: These group of medicinal plants don't have common English name

Table S2: Characteristics of pregnant women according to social drug use prior to admission to JUMC, Ethiopia

**Characteristics**	**No. (%)**	**Social drug use during pregnancy [j]**			
		No	Yes		

	1117 (100) [a]	No. (%) 1009 (90.3)	No. (%) 108 (9.7)	Crude OR [95% CI] [k]	Adjusted OR [95% CI] [l]
**Place of Residence**					
Urban	595 (53.3)	535 (53.0)	60 (55.6)	1.11 [0.74-1.65]	
Rural	522 (46.7)	474 (47.0)	48 (44.4)	1	-
**Age (years) [b]**					
≤ 20	223 (20.0)	201 (19.9)	22 (20.4)	1.59 [0.76-3.30]	1.74 [0.81-3.75]
21-25	388 (34.7)	345 (34.2)	43 (39.8)	1.81 [0.93-3.52]	1.90 [0.95-3.81]
26-30	320 (28.7)	289 (28.6)	31 (28.7)	1.56 [0.78-3.11]	1.52 [0.74-3.13]
≥ 31	186 (16.7)	174 (17.2)	12 (11.1)	1	1
**Marital status**					
Married	1071 (95.9)	967 (95.8)	104 (96.3)	1.13 [0.40-3.21]	
Others [c]	46 (4.1)	42 (4.2)	4 (3.7)	1	-
**Religion**					
Islam	731 (65.4)	662 (65.6)	69 (63.9)	2.71 [0.83-8.82]	**3.79 [1.13-12.76]**
Orthodox	305 (27.3)	269 (26.7)	36 (33.3)	**3.48 [1.04-11.60]**	**4.08 [1.19-13.99]**
Protestant/Others [d]	81 (7.3)	78 (7.7)	3 (3.7)	1	1
**Educational level [e]**					
Illiterate	378 (34.0)	344 (34.3)	34 (31.4)	0.62 [0.32-1.21]	0.66 [0.32-1.37]
Primary /read & write	470 (42.3)	435 (43.3)	35 (32.4)	**0.51 [0.26-0.98]**	0.57 [0.28-1.17]
Secondary school	162 (14.6)	137 (13.6)	25 (23.1)	1.15 [0.57-2.33]	0.99 [0.47-2.08]
Post-secondary school	102 (9.2	88 (8.8)	14 (13.0)	1	1
**Occupation**					
House wife	524 (46.9)	479 (47.5)	45 (41.7	0.68 [0.32-1.45]	
Farmer	261 (23.4)	233 (23.9)	28 (25.9)	0.87 [0.39-1.93]	
Trader/Merchant	163 (14.6)	149 (14.8)	14 (13.0)	0.68 [0.28-1.65]	
Government employee	95 (8.5)	83 (8.2)	12 (11.1)	1.04 [0.42-2.63]	
Others [f]	74 (6.6)	65 (6.4)	9 (8.3)	1	-
**Ethnic Group**					
Oromo	779 (69.7)	703 (69.7)	76 (70.4)	1.09 [0.53-2.26]	
Amhara	87 (7.8)	79 (7.8)	8 (7.4)	1.02 [0.38-2.78]	
Yem	81 (7.3)	72 (7.1)	9 (8.3)	1.26 [0.48-3.35]	
Dawuro	70 (6.3)	64 (6.3)	6 (5.6)	0.95 [0.32-2.80]	
Others [g]	100 (9.0)	91 (9.0)	9 (8.3)	1	-
**Walking distance to the nearest health facility**					
Close, ≤30 min.	731 (66.4)	662 (65.6)	69 (63.9)	0.76 [0.42-1.38]	
Somewhat far, 31-60 min.	245 (22.3)	225 (22.3)	20 (18.5)	0.65 [0.32-1.32]	
Far, >60 min.	125 (11.4)	110 (10.9)	15 (13.9)	1	-
**Gravidity [h]**					
Primigravida	431 (38.6)	392 (38.9)	39 (36.1)	0.89 [0.59-1.34]	
Multigravida	686 (61.4)	617 (61.1)	69 (63.9)	1	-
**Gestational age**					
Preterm pregnancy	231 (20.7)	192 (19.0)	39 (36.1)	1.44 [0.70-2.96]	0.88 [0.37-2.09]
Term pregnancy	735 (65.8)	683 (67.7)	52 (48.1)	0.54 [0.27-1.08]	0.59 [0.29-1.21]
Post term pregnancy	62 (5.6)	56 (5.6)	6 (2.7)	0.76 [0.27-2.18]	0.72 [0.24-2.14]
Others	89 (8.0)	78 (7.7)	11 (10.2)	1	1
**Patient type**					
Gynaecology ward	125 (11.2)	98 (9.7)	27 (25.0)	**3.10 [1.91-5.02]**	**2.81 [1.31-6.04]**
Maternity ward	992 (88.8)	911 (90.3)	81 (75.0)	1	1
**Chronic illness [i]**					
Yes	56 (5.0)	48 (4.8)	8 (7.4)	1.60 [0.74-3.48]	1.43 [0.62-3.31]
No	1061 (95.0)	961 (95.2)	100 (92.4)	1	1
**Medicinal plant use**					
Yes	319 (28.6)	268 (26.6)	51 (47.2)	**2.47 [1.65-3.70]**	**2.75 [1.79-4.24]**
No	798 (71.4	741 (73.4)	57 (52.8)	1	1
**Duration of hospital stay**					
≤ 3 days	667 (59.7)	615 (61.0)	52 (48.1)	**0.60 [0.40-0.87]**	**0.63 [0.41-0.95]**
> 3 days	450 (40.3)	394 (39.0)	56 (51.9)	1	1

[a]Numbers may not add up to 1117 due to missing values, [b]Median age 25 years, interquartile range 22–30 years; [c]Others includes single 41(3.7%), divorced 4(0.4%), widowed 1(0.1%); [d]Protestant/Others includes Protestant 74(6.6), Catholic 2(0.2%), Waqqefeta 1(0.1%), missing 4(0.4); [e]Read & write: no formal education but can read and write due to literacy campaigns, traditional religious institution and informal peer learning, Primary school: Grade 1–8, Secondary school: Grade 9–12; Post-secondary school: Technical and vocational school, college, university; [f]Others includes daily labourers 24(2.1), students 22(2.0), private institution workers 18(1.6), other sectors 10(0.9%); [g]Others includes Silte 30(2.7), Kaffa 16(1.4), Tigre 3(0.3), Wolayita 3(0.3), mixed ethnic backgrounds 7(0.6); [h]Gravidity includes the current pregnancy; [i] includes asthma, cardiac diseases, chronic gastritis/peptic ulcer, hypertension, HIV, chronic renal failure, chronic liver disease, diabetes mellitus, etc; [j]Social drug use before hospitalization; ; [k] CI, confidence interval, OR, odds ratio; Significant findings are in bold (P<0.05) [l] Adjusted for age, religion, educational level, gestational age, patient type, chronic illness, medicinal plant use and duration of hospital stay.
